# Relationships of beverage consumption and actigraphy-assessed sleep parameters among urban-dwelling youth from Mexico

**DOI:** 10.1017/S136898002100313X

**Published:** 2022-07

**Authors:** Erica C Jansen, Kathleen Corcoran, Wei Perng, Galit L Dunietz, Alejandra Cantoral, Ling Zhou, Martha M Téllez-Rojo, Karen E Peterson

**Affiliations:** 1 Department of Nutritional Sciences, University of Michigan School of Public Health, 3863 SPH I, Washington Heights, Ann Arbor, MI 48103, USA; 2 Department of Neurology, Division of Sleep Medicine, Michigan Medicine, Ann Arbor, MI, USA; 3 Department of Epidemiology, Lifecourse Epidemiology of Adiposity and Diabetes (LEAD) Center, Colorado School of Public Health, University of Colorado Denver Anschutz Medical Campus, Aurora, CO, USA; 4 CONACYT, National Institute of Public Health, Cuernavaca, Mexico; 5 Center of Statistical Research and School of Statistics, Southwestern University of Finance and Economics, Chengdu, China; 6 Center for Research on Nutrition and Health, National Institute of Public Health, Cuernavaca, Mexico; 7 Department of Environmental Health Sciences, University of Michigan School of Public Health, Ann Arbor, MI, USA

**Keywords:** Milk, Water, Soda, Fruit juice, Sleep health

## Abstract

**Objective:**

To examine whether usual beverage intake was associated with sleep timing, duration and fragmentation among adolescents.

**Design:**

Usual beverage intake was assessed with a FFQ. Outcomes included sleep duration, midpoint (median of bed and wake times) and fragmentation, assessed with 7-d actigraphy. Sex-stratified linear regression was conducted with sleep characteristics as separate outcomes and quantiles of energy-adjusted beverage intake as exposures, accounting for age, maternal education, physical activity and smoking.

**Setting:**

Mexico City.

**Participants:**

528 adolescents residing in Mexico City enrolled in a longitudinal cohort.

**Results:**

The mean age (sd) was 14·4 (2·1) years; 48 % were male. Among males, milk and water consumption were associated with longer weekday sleep duration (25 (95 % CI 1, 48) and 26 (95 % CI 4, 47) more minutes, in the 4th compared to the 1st quartile); and higher 100 % fruit juice consumption was related to earlier weekday sleep timing (−22 (95 % CI −28, 1) minutes in the 1st compared to the last quantile; *P* = 0·03). Among females, soda was associated with higher sleep fragmentation (1·6 (95 % CI 0·4, 2·8) % in the 4th compared to the 1st), and coffee/tea consumption was related to shorter weekend sleep duration (−23 (95 % CI −44, 2) minutes in the 4th compared to the 1st).

**Conclusions:**

Among females, adverse associations with sleep were observed for caffeinated drinks, while males with higher consumption of healthier beverage options (water, milk and 100 % juice) had evidence of longer and earlier-timed sleep. Potential mechanisms involving melatonin and tryptophan should be further investigated.

Insufficient sleep duration, later bedtimes and rise times, and fragmented sleep (non-continuous sleep with frequent night-time awakenings) have each been related to a myriad of health outcomes in adolescence, including changes in metabolic regulating hormones, hypertension, poorer academic performance and higher depression symptoms^(1)^. These sleep characteristics in adolescence have both biological and behavioural causes, including natural delays in sleep onset (circadian phase shift), early school start times, academic and extracurricular pressures, and use of social media and screens late at night^(1)^.

The intake of caffeinated, sugar-sweetened beverages is a common and modifiable behaviour among adolescents that has the potential to impact sleep (in addition to being affected by sleep^(2)^). In particular, higher consumption of sugar-sweetened beverages and/or caffeine among adolescents has been linked to poor sleep quality, including shorter sleep duration^(3–6)^, later bedtimes^(4)^ and larger variability in sleep duration^(6)^. Further, these associations may be sex-specific; one study among adolescents with overweight/obesity reported an association between weekend wake time and sugar-sweetened beverage consumption among males but not females^(7)^. However, sugar-sweetened beverages are just one class of beverages that adolescents may consume on a daily basis, and limited investigations on the effects of other beverages exist. Of the few publications examining non-sugar-sweetened beverage consumption and sleep in adolescents, one twelve-country study reported that longer sleep duration was associated with higher consumption of fruit juice (although not specified whether 100 % juice or not)^(3)^, while another US study^(4)^ found no association between sleep duration and 100 % juice (no added sugar) intake. Despite the lack of adolescent evidence, prior experimental research among certain populations of adults suggests that higher milk and tart cherry juice consumption are related to higher quality sleep, potentially mediated through increases in tryptophan and melatonin^(8–10)^. Tryptophan is an amino acid that is a precursor to melatonin, the sleep/wake hormone that plays an important role in sleep onset and continuity. Our aim was to examine associations of usual beverage intake in relation to sleep timing, duration and fragmentation for the following groups of beverages: milk, sweetened milk, regular soda, coffee/tea, 100 % fruit juice, commercial fruit juice with sugar, fruit drink/flavoured water with sugar and plain water. We hypothesized that intake of milk, 100 % juice and water would be associated with better sleep quality (i.e. longer duration, earlier bedtimes and rise times^(11)^, and less fragmentation), while intake of soda and coffee/tea (which contain added sugar and typically caffeine) would be associated with poor sleep quality among the adolescents in our sample. Specific hypotheses regarding the other types of beverages were not made and were considered as exploratory.

## Participants and methods

The study sample included participants from two of three sequentially enrolled cohorts of the Early Life Exposure in Mexico to ENvironmental Toxicants (ELEMENT) study^(12)^. Between 1997 and 2004, 1012 mother–child dyads were recruited by trained research assistants from prenatal clinics of the Mexican Social Security Institute in Mexico City. These clinics serve low- to middle-income populations formally employed in the private sector; thus, our study population is considered to reflect this segment of the population. At baseline, mothers reported socio-demographic and health information. In 2015, a subset of 550 participants from the original birth cohorts 2 and 3 (which included 1079 children overall), who were presumed to be in the midst of the pubertal transition, agreed to participate in a follow-up visit. The adolescents received a set of headphones, and their families received a grocery voucher for their participation. All of the 550 participants who attended the clinical visit responded to the FFQ and 539 provided actigraphy data. After excluding 11 participants with incomplete actigraph information (<4 d of wear time), the analytic sample included 528 participants aged 9 to 17 years. The WHO defines adolescence as the period from 10 to 19 years of age; since only two participants in our analytic sample were 9 years old (ages 9·8 and 9·9 years), we henceforth refer to this study population as an adolescent sample. The study was conducted according to the guidelines laid down in the Declaration of Helsinki, and all procedures involving research study participants were approved by the institutional review boards at the Mexico National Institute of Public Health and the University of Michigan. Written informed consent was obtained from parents for all participants in addition to participant assent.

### Beverage intake

During the clinic visit, a trained social worker administered a semi-quantitative FFQ with visual aids of food items and portion sizes to adolescents who were often assisted by mothers. This questionnaire has been evaluated in a Mexican population by comparing with 24-h recalls (deattenuated correlation coefficients of nutrients ranging from 0·19 (Zn) to 0·61 (Ca) for adolescents)^(13)^. Briefly, the semi-quantitative FFQ asked the adolescents to recall how often in the previous 7 d they consumed each food or beverage item from a list of 116 common foods, including 11 different types of beverages. Possible responses to the frequency of food and beverages consumption ranged from never to more than six times/d. Additional information was collected about approximate portion sizes of each consumed item. With the use of nutrient composition tables, average quantity of beverage consumption was calculated (ml/d) and average total energetic intake was computed. Caffeine intake was also estimated based on the assumption that most regular sodas and coffee/tea were caffeinated (the FFQ did not differentiate caffeinated from non-caffeinated beverages). Based on nutritional similarity, beverages were grouped into eight categories: milk, sweetened milk, regular soda, coffee/tea, 100 % fruit juice, commercial fruit juice with sugar, fruit drink/flavoured water with sugar and plain water. All beverages (including water) were adjusted for total energy intake using the residual method^(14)^. In the residual method, beverage consumption (in ml) is regressed on total energies, and the residuals of this model then represent whether the beverage intake is relatively higher (positive values) or lower (negative values) after accounting for the expected intake due to the overall energetic consumption. The residual values were added to the population-level mean intakes of beverages for improved interpretability.

### Sleep measures

At the end of the clinical follow-up visit, adolescents were given an actigraph (ActiGraph GT3X+; ActiGraph LLC, Pensacola, FL) to wear on their non-dominant wrist continuously for 7 d. Nightly sleep parameters were estimated from the actigraphic data with the use of a fusedLASSO (least absolute shrinkage and selection operator)-based calculator package developed in R (R Foundation for Statistical Computing). As primary endpoints of the study, we obtained weekday and weekend sleep duration (minutes), weekday and weekend sleep midpoint (the median of sleep onset and wake time, reported in decimal hours), and average sleep fragmentation index. Sleep fragmentation index, a standard sleep parameter computed by Actilife® software^(15)^, was calculated as the percentage of 1-min (or shorter) periods of sleep out of the total number of sleep bouts of any length^(16)^, with higher values representing more fragmented sleep. The three sleep characteristics were used as separate outcomes and each represents a unique aspect of sleep that is independently associated with health outcomes. Sleep duration provides an indication regarding whether a recommended amount of sleep is obtained, while sleep midpoint is a marker of whether the adolescent has a sleep schedule that may be misaligned with internal circadian rhythms (circadian misalignment), and sleep fragmentation gives information on the continuity of sleep.

### Covariates

Possible confounders were selected a priori due to reported associations with sleep and/or beverage consumption among adolescents^(1,17,18)^ and included sex, age, BMI-for-age *Z*-scores^(19)^, maternal education, physical activity, screen time and smoking status (ever/never). Trained research assistants measured height in cm (BAME Model 420; Catalogo Medico) and weight in kg (BAME Model 420; Catalogo Medico). BMI *Z*-scores (BMIz) were calculated based on the WHO reference^(19)^. Maternal education was reported by mothers at the original cohort enrolment visit and considered as a proxy for socio-economic status. Physical activity and screen time were assessed with a questionnaire adapted for and evaluated against 24-h recalls of physical activity in Mexican adolescents^(20)^. Pubertal status, assessed by Tanner staging and testicular volume assessment (for males), was completed by trained physicians during the visit using standard methods^(21)^. Females were also asked about menarche.

### Statistical analysis

First, associations between covariates and beverage consumption were examined by comparing the mean and standard deviation of energy-adjusted beverage intake (ml/d, separately for each beverage category) across categories of covariates. Spearman’s correlations of energy-adjusted beverage intakes were also computed to evaluate the extent to which consumption of individual beverages may be associated with others (and thus potential confounders). Next, bivariate associations between energy-adjusted beverage intakes and sleep measures were evaluated by estimating the means and standard devaition of sleep duration (separately for weekday *v*. weekend), midpoint (separately for weekday *v*. weekend) and fragmentation index, according to quartiles of each beverage (tertiles or dichotomously for beverages with a high proportion of non-consumers). In sex-stratified multivariable analysis, separate linear regression models were run with continuous sleep measures (sleep duration, midpoint and fragmentation index) as the outcome and indicator variables for categories of beverage intake as the exposure (each beverage was run in its own model), adjusting for age, maternal education, physical activity and smoking. Pubertal stage was highly correlated with age and thus was not included in the final models. BMIz was considered as a potential mediator (i.e. more likely caused by differences in beverage intakes, particularly sugar-sweetened beverages, rather than the other way around) and not included as a confounder. *P*-values to assess linear relationships were obtained from linear regression models with sleep measures as the outcome and a variable representing ordinal categories of each beverage (i.e. four categories for quartiles and three categories for tertiles). Several *post hoc* analyses were also run. First, we ran models that examined beverages as a dichotomous variable for daily consumers *v*. non-daily consumers. Due to low frequency of intake in some beverages, we conducted these models only for water, soda and milk. Second, we ran models that examined caffeine intake as a continuous predictor. Third, models were run with mutual adjustment for correlated beverages (i.e. we included in the same model all beverages that were statistically significantly correlated with one another but not strongly enough for collinearity to be an issue). Fourth, for beverages that were associated with BMI, models were additionally adjusted for BMI. Fifth, models that included other dietary sources of tryptophan, a sleep-promoting amino acid, were also run to assess the extent to which these sources accounted for the relationship of interest. The tryptophan food sources that were assessed included chicken, eggs, high-fat dairy and processed meat. These items were selected due to a high tryptophan content as well as being commonly consumed within this population. Finally, for the main findings, we examined whether associations depended on the season of actigraphy assessment models (data collected in June, July and August *v*. data collected during the school year) by conducting interaction analyses between beverages and season of collection. All analyses were conducted in Stata 14.0. Statistical testing adjustments for multiple comparisons were not made due to the fact that both the exposures and the outcomes were correlated.

## Results

The mean age (sd) of the study population was 14·4 (2·1) years with a range of 9·8 to 18·1 years; 48 % were male. The most commonly consumed drinks were (in order) water, milk, soda and juice drink (approximate energy-adjusted servings/d were 2·7, 1·4, 0·9 and 0·8 servings/d, respectively). Associations of energy-adjusted beverage intake with socio-demographic and lifestyle characteristics are found in Table 1. Males consumed more soda and water than females. Milk consumption was inversely associated with age, while coffee consumption was positively associated with age. Juice drink was non-linearly correlated with age, with the highest consumption among 12 to <14 year olds and the lowest consumption among 14 to <16 year olds. Adolescents with a BMI-for-age *Z*-score >2 reported higher water consumption, and adolescents whose mothers had higher education had lower consumption of soda but higher consumption of juice drink. The lowest intake of soda and highest intake of water were found among those with the highest weekly self-reported physical activity. Screen time was positively associated with soda consumption, while ever having tried smoking was associated with drinking less milk and juice drink but more soda.

**Table 1:** Energy-adjusted beverage intake of 528 youth aged 9–18 years from Mexico City, according to socio-demographic and lifestyle characteristics

Average intake per d (sd), in energy-adjusted ml																	
Socio-demographic and lifestyle characteristics	*n*	Milk		Sweetened milk		Regular soda		Coffee and tea		100 % juice		Juice drink		Flavoured water withsugar		Water	
		Mean	sd	Mean	sd	Mean	sd	Mean	sd	Mean	sd	Mean	sd	Mean	sd	Mean	sd
Sex																	
Male	252	397	297	3	19	253	263	66	113	29	58	217	279	78	194	718	598
Female	276	336	259	5	29	199	237	77	120	28	61	190	247	89	209	618	513
*P*-value		0·06		0·67		0·009		0·12		0·97		0·51		0·53		0·05	
Age group																	
9·5 to <12 years	93	447	269	5	26	180	199	36	66	28	55	234	321	73	179	738	627
12 to < 14 years	154	400	311	5	27	205	226	68	107	33	68	260	266	88	233	605	521
14 to < 16 years	98	319	282	3	19	254	307	85	136	23	56	150	229	103	204	652	560
16 to 18 years	183	305	235	4	25	249	259	86	130	28	56	168	234	75	183	687	544
*P*-value		0·0001		0·73		0·28		0·003		0·50		0·0001		0·14		0·49	
Pubertal status*																	
Earlier stages	101	428	289	4	22	253	263	56	103	21	46	179	264	107	246	629	546
Latter stages	427	344	274	4	25	199	237	75	119	30	62	209	263	78	189	674	560
*P*-value		0·007		0·84		0·73		0·06		0·11		0·39		0·04		0·45	
BMI-for-age *Z*-score†																	
< 0	54	352	243	8	38	218	189	56	84	21	44	136	173	62	119	638	591
0 to < 1	129	358	252	3	23	208	221	82	129	32	76	201	242	86	213	605	551
1 to < 2	141	342	287	2	12	245	296	73	106	26	55	194	237	91	226	571	491
≥ 2	202	379	298	6	28	226	251	66	119	28	53	225	305	84	195	780	580
*P*-value		0·65		0·64		0·82		0·41		0·81		0·52		0·97		0·005	
Maternal education, years																	
8 years or less	61	292	253	5	30	244	223	62	103	32	58	145	235	69	134	643	621
9 to 11 years	205	360	284	5	27	242	240	74	122	24	54	195	263	85	208	637	525
12 years	181	368	289	4	23	231	284	80	126	27	54	192	249	103	232	696	557
>12 years	76	397	256	1	4	152	211	54	92	41	84	303	300	44	145	715	595
*P*-value		0·09		0·54		0·002		0·63		0·40		0·0007		0·09		0·61	
Physical activity, quartiles (h/wk)																	
Q1, 0 to 5·5	136	344	281	7	34	279	298	63	99	19	47	191	284	111	267	567	533
Q2, 5·8 to 9	128	357	287	4	21	218	211	78	125	23	43	189	233	64	134	615	498
Q3, 9·3 to 14	138	379	252	4	23	226	270	70	119	30	56	201	247	77	194	728	587
Q4, 14·3 to 29	126	360	297	2	17	172	196	76	124	42	83	234	287	81	185	754	588
*P*-value		0·44		0·83		0·01		0·91		0·05		0·33		0·84		0·01	
Screen time, quartiles (h/wk)																	
Q1, 1 to 22·5	135	369	265	4	24	194	220	55	86	24	51	208	261	61	152	649	500
Q2, 23 to 32·5	130	370	260	3	14	215	243	68	109	30	60	226	289	86	210	721	634
Q3, 33 to 48	131	365	302	8	37	222	277	84	140	28	68	195	278	97	252	672	561
Q4, 48·5 to 116	132	338	288	3	20	270	258	80	125	31	59	183	221	91	180	622	527
*P*-value		0·54		0·42		0·04		0·46		0·61		0·56		0·72		0·69	
Ever smoked cigarettes																	
No	391	384	287	5	28	197	216	64	105	29	59	216	272	83	205	664	552
Yes	133	298	242	2	12	304	322	93	144	28	63	164	230	87	194	667	577
*P*-value		0·002		0·66		0·0007		0·05		0·79		0·03		0·84		0·80	

ml = millilitres, h/wk = hours per week.

* Earlier and latter stages of puberty divided by having started menarche (latter stage) for females and testicular volume >15 ml for males (latter).

† Based on WHO reference.

Inverse correlations existed between intake of milk and soda (−0·15), milk and coffee/tea (−0·23), and water and soda (−0·20, Table 2). Similarly, there was an inverse correlation between flavoured water with added sugar and juice drink (−0·11), while there was a positive correlation between water and natural fruit juice (0·13).

**Table 2:** Spearman’s correlations between energy-adjusted beverage intakes in a sample of 528 youth aged 9–18 years from Mexico City

	Milk	Sweetened milk	Regular soda	Coffee and tea	Natural fruit juice	Fruit juice with added sugar	Flavoured water with sugar
Milk	1·0000						
Sweetened milk	−0·0584	1·0000					
Regular soda	−0·1473*	0·0227	1·0000				
Coffee and tea	−0·2322*	−0·0531	−0·0587	1·0000			
Natural fruit juice	0·0171	0·0449	−0·0527	−0·0702	1·0000		
Fruit juice with added sugar	−0·0037	−0·0107	−0·1043*	−0·0791	0·1020	1·0000	
Flavoured water with sugar	−0·0488	−0·0159	0·0553	−0·0060	−0·0805*	−0·1090*	1·0000
Water	0·0860*	0·0711	−0·2047*	−0·0182	0·1285*	−0·0254	−0·0747

* *P* < 0·05.

Average weekday sleep duration was 8 h, 25 min ± 76 min/d, with 41 % of participants meeting sleep duration recommendations for their age^(22)^. Average weekend sleep duration was 9 h, 3 min ± 79 min/d. The average weekday bedtime was 11:40 PM ± 1·3 h and average wake time was 8:04 AM ± 1·8 h. Thus, the average weekday sleep midpoint (median of bed and wake times) was 3·9 ± 1·4 h (i.e. 3:54 AM ± 84 min), while on the weekend it was 4·8 ± 1·3 h (4:48 AM ± 78 min).

Among males, weekly reporting of water consumption was associated with longer weekday sleep duration after accounting for the potential confounders of age, maternal education, screen time and physical activity (26 (95 % CI 4, 47) more minutes in the 4th compared to the 1st quartile, Table 3; additional adjustment for BMI did not alter estimates). Weekly reporting of milk was also associated with longer sleep duration (25 (95 % CI 1, 48) more minutes in the 4th compared to the 1st quartile). Further, daily consumers of milk had on average 19 more minutes of sleep compared to non-daily consumers (95 % CI 2·7, 36·0; *P* = 0·02). In addition, higher weekly reporting of 100 % fruit juice consumption was related to earlier weekday sleep timing (−22 (95 % CI −28, 1) minutes in the 1st compared to the last quantile; *P* = 0·03), an association that was reflective of both earlier bedtimes and earlier wake times in the higher consumers (not shown).

**Table 3:** Associations between beverage intake and actigraphy-assessed sleep characteristics in 252 males aged 9–18 years from Mexico City

	*n*	Weekday duration (minutes)				Weekday midpoint (decimal hour)				Total fragmentation index (%)			
		Mean	sd	Adjusted difference	95 % CI†	Mean	sd	Adjusted difference	95 % CI	Mean	sd	Adjusted difference	95 % CI
Milk													
Q1 (mean = 69 ml)	57	493	73	Ref		4·25	1·39	Ref		11·5	4·1	Ref	
Q2 (mean = 211 ml)	60	505	77	15	−8, 38	4·09	1·66	−0·22	−0·68, 0·23	13·1	4·2	2·1	0·7, 3·5
Q3 (mean = 439 ml)	69	494	69	13	−10, 36	3·76	1·33	−0·38	−0·83, 0·06	12·3	3·4	1·3	−0·1, 2·7
Q4 (mean = 766 ml)	66	518	61	25	1, 48*	3·97	1·39	−0·19	−0·64, 0·27	12·1	3·8	1·3	−0·1, 2·7
*P*-value‡				0·06				0·34				0·20	
Sweetened milk§													
Q1 (mean = 0 ml)	242	501	70	Ref		3·98	1·46	Ref		12·3	3·9	Ref	
Q2 (mean = 85 ml)	10	545	67	25	−16, 65	4·55	0·65	0·27	−0·51, 1·06	12·1	3·1	−0·3	−2·8, 2·2
*P*-value				0·24				0·50				0·80	
Regular soda													
Q1 (mean = 9 ml)	55	507	80	Ref		3·78	1·29	Ref		12·4	3·7	Ref	
Q2 (mean = 94 ml)	58	500	70	−15	−39, 8	3·97	1·29	0·29	−0·16, 0·75	11·7	4·0	−0·7	−2·2, 0·7
Q3 (mean = 227 ml)	64	496	62	−19	−42, 5	3·79	1·35	0·17	−0·29, 0·62	12·6	4·1	0·4	−1·1, 1·8
Q4 (mean = 578 ml)	75	507	71	−11	−34, 12	4·38	1·67	0·40	−0·04, 0·84	12·3	3·8	−0·4	−1·8, 1·0
*P*-value				0·40				0·13				0·96	
Coffee and tea‖													
Q1 (mean = 0 ml)	132	499	69	Ref		3·84	1·43	Ref		12·4	3·6	Ref	
Q2 (mean = 60 ml)	68	512	67	8	−11, 27	4·18	1·44	0·17	−0·20, 0·53	12·2	4·2	−0·3	−1·5, 0·8
Q3 (mean = 242 ml)	52	500	77	−2	−24, 19	4·19	1·47	0·06	−0·36, 0·47	12·0	4·2	−0·3	−1·6, 1·0
*P*-value				0·97				0·62				0·57	
Natural fruit juice‖													
Q1 (mean = 0 ml)	173	505	74	Ref		4·16	1·49	Ref		12·4	4·0	Ref	
Q2 (mean = 39 ml)	35	495	67	3	−21, 27	3·61	1·08	−0·48	−0·93, −0·04*	12·5	4·0	−0·04	−1·5, 1·4
Q3 (mean = 133 ml)	44	500	56	5	−17, 26	3·70	1·45	−0·37	−0·78, 0·04	11·5	3·5	−1·1	−2·4, 0·2
*P*-value				0·65				0·03				0·12	
Fruit juice with added sugar													
Q0 (mean = 0 ml)	93	506	64	Ref		4·09	1·48	Ref		12·2	3·8	Ref	
Q1 (mean = 80 ml)	50	498	83	1	−21, 23	3·65	1·30	−0·30	−0·72, 0·13	11·6	3·6	−0·5	−1·8, 0·9
Q2 (mean = 257 ml)	50	500	64	−4	−26, 18	4·07	1·37	0·09	−0·33, 0·52	12·6	3·7	0·5	−0·9, 1·8
Q3 (mean = 643 ml)	59	505	74	−3	−25, 18	4·12	1·56	0·16	−0·25, 0·57	12·4	4·5	0·5	−0·8, 1·9
*P*-value				0·70				0·31				0·30	
Flavoured water with sugar‖													
Q1 (mean = 0 ml)	185	501	70	Ref		4·05	1·43	Ref		12·3	3·9	Ref	
Q2 (mean = 115 ml)	38	503	70	4	−19, 27	3·91	1·35	−0·006	−0·45, 0·44	12·4	3·7	0·3	−1·1, 1·7
Q3 (mean = 524 ml)	29	514	71	12	−14, 37	3·84	1·66	−0·12	−0·61, 0·37	12·0	4·0	−0·1	−1·6, 1·5
*P*-value				0·36				0·67				0·94	
Water													
Q1 (mean = 68 ml)	62	497	72	Ref		4·11	1·56	Ref		12·9	4·0	Ref	
Q2 (mean = 375 ml)	54	495	75	−5	−28, 19	4·04	1·50	0·04	−0·42, 0·49	12·2	4·2	−0·6	−2·0, 0·9
Q3 (mean = 779 ml)	58	498	72	1	−22, 24	4·04	1·38	0·01	−0·44, 0·46	11·6	3·6	−1·3	−2·7, 0·1
Q4 (mean = 1425 ml)	78	516	63	26	4, 47*	3·87	1·37	−0·32	−0·74, 0·11	12·3	3·8	−0·4	−1·7, 1·0
*P*-value				0·02				0·14				0·46	

* Statistically significantly different at *P* < 0·05.

† From linear regression models with sleep characteristic as the continuous outcome and indicator variables for quantiles of beverage intake as the predictor, adjusted for age, maternal education, screen time and physical activity.

‡ *P*_for trends_ are from a Wald test of a continuous variable representing ordinal categories of beverage intake quantile.

§ Divided into a dichotomous variable due to high proportion of non-consumers.

‖ Divided into tertiles rather than quartiles due to high proportion of non-consumers.

Among females only, higher weekly reporting of soda consumption was associated with higher sleep fragmentation (1·6 (95 % CI 0·4, 2·8) % in the 4th compared to the 1st, Table 4; and 1·8 with (95 % CI 0·5, 3·0) in daily consumers *v*. non-daily consumers), although this association was only found during the school year (*P*_for interaction_ of school year *v*. summer = 0·005). In addition, coffee/tea consumption was related to shorter weekend sleep duration (−23 (95 % CI −44, 2) minutes in the 4th compared to the 1st; see online Supplemental Table 1). Finally, daily water consumers had a later sleep midpoint than non-daily water consumers (0·36 h with 95 % CI 0·06, 0·67; *P* = 0·02). In sensitivity analysis where correlated beverages were included in the same models (e.g. soda and water), findings were unaltered. Similarly, additional adjustment for dietary sources of tryptophan (chicken, eggs, high-fat dairy and processed meat) did not alter the findings. Further, associations held after excluding actigraphy data collected during the summer. In *post hoc* analyses that examined estimated caffeine intake in relation to sleep, there was no association.

**Table 4:** Associations between beverage intake and actigraphy-assessed sleep characteristics in 276 females aged 9–18 years from Mexico City

	*n*	Weekday duration (minutes)				Weekday midpoint (decimal hour)				Total fragmentation index (%)			
		Mean	sd	Adjusted Difference	95 % CI†	Mean	sd	Adjusted difference	95 % CI	Mean	sd	Adjusted difference	95 % CI
Milk													
Q1 (mean = 61 ml)	75	501	92	Ref		3·65	1·25	Ref		11·7	2·8	Ref	
Q2 (mean = 215 ml)	72	510	82	−5	−30, 20	4·08	1·43	0·39	0·004, 0·78	11·0	3·5	−0·6	−1·7, 0·5
Q3 (mean = 412 ml)	63	499	68	−2	−27, 23	3·60	1·27	0·06	−0·34, 0·45	11·2	3·3	−0·3	−1·5, 0·8
Q4 (mean = 709 ml)	66	521	80	15	−10, 40	3·65	1·42	0·01	−0·39, 0·41	11·4	3·6	0·0	−1·1, 1·1
*P*-value‡				0·25				0·68				0·92	
Sweetened milk§													
Q1 (mean = 0 ml)	263	508	80	Ref		3·78	1·36	Ref		11·4	3·3	Ref	
Q2 (mean = 112 ml)	13	500	111	−7	−12, −2	3·24	1·12	−0·38	−1·06, 0·30	10·4	2·7	−0·8	−2·7, 1·2
*P*-value				0·63				0·28				0·44	
Regular soda													
Q1 (mean = 10 ml)	77	494	90	Ref		3·57	1·30	Ref		11·1	3·1	Ref	
Q2 (mean = 93 ml)	74	504	77	5	−19, 29	3·62	1·44	0·08	−0·30, 0·46	10·9	2·7	−0·2	−1·2, 0·9
Q3 (mean = 224 ml)	68	511	74	4	−21, 29	3·85	1·31	0·31	−0·08, 0·71	11·1	3·5	0·0	−1·1, 1·1
Q4 (mean = 563 ml)	57	526	83	19	−8, 46	4·05	1·31	0·25	−0·18, 0·69	12·6	3·7	1·6	0·4, 2·8*
*P*-value				0·23				0·13				0·02	
Coffee and tea‖													
Q1 (mean = 0 ml)	131	511	82	Ref		3·66	1·40	Ref		11·4	3·6	Ref	
Q2 (mean = 58 ml)	65	509	69	−5	−27, 18	3·69	1·34	0·12	−0·23, 0·47	11·2	2·8	−0·3	−1·3, 0·7
Q3 (mean = 218 ml)	80	500	91	−15	−36, 6	3·95	1·27	0·30	−0·03, 0·63	11·2	3·1	−0·3	−1·2, 0·7
*P*-value				0·16				0·08				0·55	
Natural fruit juice‖													
Q1 (mean = 0 ml)	188	508	82	Ref		3·76	1·40	Ref		11·3	3·5	Ref	
Q2 (mean = 41 ml)	49	507	76	4	−20, 27	3·58	1·28	−0·18	−0·55, 0·20	11·8	2·7	0·5	−0·5, 1·6
Q3 (mean = 148 ml)	39	504	87	−7	−33, 19	3·93	1·21	0·15	−0·26, 0·56	10·8	3·0	−0·6	−1·8, 0·6
*P*-value				0·71				0·76				0·58	
Fruit juice with added sugar													
Q1 (mean = 0 ml)	102	506	85	Ref		3·75	1·47	Ref		11·3	3·3	Ref	
Q2 (mean = 88 ml)	61	493	87	−15	−39, 9	3·70	1·30	0·25	−0·13, 0·63	10·9	3·2	−0·2	−1·3, 0·9
Q3 (mean = 61 ml)	61	523	74	5	−19, 29	4·10	1·32	0·37	−0·01, 0·75	11·6	3·6	0·5	−0·6, 1·6
Q4 (mean = 53 ml)	52	509	76	11	−15, 37	3·41	1·11	−0·22	−0·62, 0·19	11·5	3·1	0·4	−0·7, 1·6
*P*-value				0·29				0·75				0·28	
Flavoured water with sugar‖													
Q1 (mean = 0 ml)	197	504	78	Ref		3·72	1·40	Ref		11·4	3·3	Ref	
Q2 (mean = 106 ml)	35	501	92	−6	−33, 20	3·61	1·06	0·11	−0·32, 0·54	11·3	3·4	−0·1	−1·4, 1·1
Q3 (mean = 474 ml)	44	528	87	19	−5, 44	4·00	1·32	0·07	−0·33, 0·46	10·9	3·1	−0·7	−1·9, 0·4
*P*-value				0·21				0·66				0·22	
Water													
Q1 (mean = 68 ml)	70	509	83	Ref		3·61	1·24	Ref		11·1	3·5	Ref	
Q2 (mean = 408 ml)	78	513	76	5	−19, 29	3·58	1·25	−0·12	−0·50, 0·26	11·4	3·4	0·3	−0·8, 1·4
Q3 (mean = 787 ml)	74	510	88	−10	−35, 14	4·10	1·50	0·36	−0·03, 0·75	11·4	3·3	0·1	−1·0, 1·2
Q4 (mean = 1402 ml)	54	493	78	−16	−43, 10	3·71	1·36	0·17	−0·25, 0·59	11·3	3·0	0·1	−1·1, 1·3
*P*-value				0·12				0·10				0·98	

* Statistically significantly different at *P* < 0·05.

† From linear regression models with sleep characteristic as the continuous outcome and indicator variables for quantiles of beverage intake as the predictor, adjusted for age, maternal education, screen time and physical activity.

‡ *P*_for trends_ are from a Wald test of a continuous variable representing ordinal categories of beverage intake quantile.

§ Divided into a dichotomous variable due to high proportion of non-consumers.

‖ Divided into tertiles rather than quartiles due to high proportion of non-consumers.

## Discussion

In this cohort of adolescents residing in Mexico City from low- to middle-income families, intake of specific beverages was cross-sectionally related to sleep in a sex-specific manner. Among females, higher weekly coffee/tea and soda consumption were associated with shorter sleep duration and higher fragmentation, respectively. Among males, higher consumption of water, milk and 100 % juice had healthier sleep parameters (longer duration, earlier midpoint and/or less fragmented sleep). Many of our findings are of clinical relevance when comparing highest beverage consumers *v*. lowest consumers, as a difference of about 30 min of total sleep time has been related to apparent differences in cognitive outcomes and mood among adolescents^(23,24)^. Overall, findings add to the existing literature on this topic by suggesting that not only sugar-sweetened beverages, but also other beverages could affect sleep patterns of adolescents. These data may be relevant to clinicians who encounter adolescents with sleep difficulties, as they highlight the role that lifestyle behaviours, including beverage consumption, could play on achieving adequate sleep duration and quality.

Among females, coffee and tea consumption were related to shorter sleep duration on weekends. In addition, soda consumption was related to higher sleep fragmentation, although the magnitude of this association was relatively small. These associations have biological plausibility through their caffeine content, since caffeine is known to interfere with adenosine, a neurotransmitter that promotes sleep^(25)^. Caffeine has also been related to worse sleep quality and to nocturnal awakenings^(26)^. The fact that caffeine-containing beverages were related to sleep only among females could be attributed to different physiological responses to caffeine in males *v*. females. For example, although sleep was not assessed, one laboratory study among adolescents showed that females had greater changes in blood pressure in response to caffeine than males, but lower changes in heart rate^(27)^. It could also be related to differences in the timing of caffeine consumption among females than males, with females perhaps consuming more caffeine in the afternoon and evening. It is important to point out that overall estimated caffeine intake was not associated with sleep within the sample, so there may be other constituents in coffee, tea and soda that are responsible for the observed associations. Nonetheless, the analyses with caffeine intake included caffeine from all sources (e.g. chocolate), and the estimated caffeine intake may have suffered from measurement error since the FFQ did not differentiate caffeinated from non-caffeinated beverages.

Although soda intake was related to later sleep midpoint in both males and females, it was not statistically significantly associated after adjusting for all the potential confounders. There were several strong potential confounders in this cohort. For example, soda consumption was highest among adolescents of lower SES (proxied by mothers with lower education), and it was also correlated with more unhealthy behaviours that often co-occur with short sleep duration and poor quality sleep, including less physical activity, more screen time and more smoking. Confounding due to SES could also have to do with the overall neighbourhood environment, for example, lower-income neighbourhoods may have greater accessibility to soda than higher-income neighbhorhoods^(28)^ as well as greater barriers to healthy sleep (e.g. noise, threats of violence). In addition to confounding, another reason that soda was not associated with later sleep timing could have to do with high levels of soda consumption overall in this population (i.e. not enough variability within soda intake to detect differences). Mexico is one of the highest consumers of soda worldwide and despite decreases in consumption following the implementation of a 2014 tax on sugar-sweetened beverages, daily consumption levels remain high^(29)^.

The finding that higher milk consumption was associated with longer sleep duration among males is in line with some prior experimental studies which have suggested that malted milk or melatonin-enriched milk consumed before bedtime may be related to lower sleep disturbances^(8)^. Although the milk consumed by children in our study population was not likely malted or enhanced with melatonin, milk naturally contains the amino acid tryptophan, which has been related to sleep duration in other epidemiological investigations^(30)^. Indeed, the daily milk consumers in our population had higher tryptophan intake (from all sources) than those who did not consume milk daily (an average of 710 ± 313 mg/d compared to 633 ± 272 mg/d; *P*, difference = 0·003). This makes sense given that there is an estimated 50 mg of tryptophan per 100 ml of whole milk^(31)^. Within the brain, tryptophan is converted via serotonin to the hormone melatonin, a major hormone responsible for regulating sleep and the circadian rhythm. In order for tryptophan to be converted to serotonin, it must successfully cross the blood–brain barrier, which is hindered when tryptophan has to compete with the other large neutral amino acids (LNAA) found in dietary protein. Typically, tryptophan is the least abundant amino acid in high-protein foods and therefore must compete with other LNAA to cross the blood–brain barrier. However, relative to other protein sources, such as pork, turkey breast, beef, chicken breast, eggs and cheese, milk has a higher tryptophan to LNAA ratio^(30)^. Furthermore, milk is also a source of carbohydrates, and the consumption of carbohydrates aids the crossing of tryptophan. The reason is that the release of insulin signals a preferential uptake of other LNAA in muscle, lending an increased likelihood for tryptophan to cross the blood–brain barrier as the concentration of other LNAA decreases^(7)^. Although milk consumption has biologic plausibility in relation to sleep, we do not have information on what time of day the milk was consumed, which could play a large role in whether or not this mechanism applies.

The finding that 100 % fruit juice intake was related to earlier sleep timing among males is novel in an adolescent population, but in line with a cross-sectional report on a positive association between overall fruit juice consumption and adherence to sleep duration recommendations^(3)^. In addition, supplementation with tart cherry juice (a natural fruit juice) in randomised trials among adults has been shown to improve sleep timing^(9,10)^ and quality^(10)^. A possible mechanism linking 100 % fruit juice and sleep timing could be related to its anti-inflammatory potential, which has been related to higher sleep quality in some studies^(32,33)^. The consumption of 100 % fruit juice could also be an indicator of an overall healthier lifestyle.

Males with higher weekly water consumption had longer weekday sleep duration. In the Mexican adolescent population, water consumption could be considered a proxy for higher physical activity^(17)^, and higher physical activity is associated with better quality of sleep^(34)^. However, the association persisted after accounting for self-reported physical activity. We also cannot preclude the possibility (both for water and for findings with the other beverages) that the direction of the association is reversed; that is, that males with longer typical sleep duration consumed more water.

We found highly sex-specific relationships between beverage intake and sleep among adolescents in our study. These sex differences have biological plausibility, given that the cohort was in the midst of the pubertal transition, and that sex steroids can affect sleep differently (i.e. oestrogen has different effects compared to testosterone)^(35)^. Sex differences in reporting of beverage intakes is also worth consideration as an alternative explanation. It is possible that adolescent females and males are affected by social desirability bias (providing responses that are perceived as more favourable) differently. For example, research among adults has shown the social desirability biases may cause men to over-report particular nutrients and total energy intakes, while women may tend to underestimate total energy intake^(36)^. Nonetheless, these same sex-specific biases have not been consistently observed in adolescents^(37)^, and the social desirability biases specifically regarding beverage consumption among adolescents remain unknown.

Strengths of this study include the use of actigraphy to assess sleep characteristics, objectively measured anthropometry and the inclusion of a wide range of beverages. Limitations include lack of information on the timing of beverage consumption (including the time of day as well as the day of the week) and timing of other behavioural determinants of poor sleep, such as screen usage at night. The FFQ did not specify whether drinks were caffeinated or decaffeinated, which hindered the ability to estimate true caffeine intake. Also, the previous validation study of the FFQ examined overall nutrients rather than individual foods, so it is difficult to ascertain how well the FFQ specifically measured drinks. Further, the cross-sectional nature of the study did not allow us to disentangle the temporal sequence of beverage consumption and sleep characteristics. The relatively wide age range of the sample likely means that sleep behaviours and norms (e.g. bedtime rules) were not homogeneous, although all analyses were adjusted for age. Given the large number of analyses conducted and the exploratory nature of the analyses, the potential for spurious findings exists. Finally, the generalisability of study results may also be limited to adolescents residing in Mexico City.

## Conclusion

In summary, this cross-sectional study found that caffeinated beverages (soda and coffee/tea) were associated with worse sleep parameters in females, while water, milk and 100 % fruit juice were associated with healthier sleep among males only. Although additional research is needed, especially regarding the timing of beverage consumption relative to bedtimes, the present findings may help to craft recommendations on beverages that promote healthy sleep in adolescents.

## Supplementary material

For supplementary material accompanying this paper visit https://doi.org/10.1017/S136898002100313X. click here to view supplementary material
