# Supplementary material for: Relationships of beverage consumption and actigraphy-assessed sleep parameters among urban-dwelling youth from Mexico
Source: Public Health Nutr. 2021 Jul 30;25(7):1844–53. doi: 10.1017/S136898002100313X (PMC8800947; doi:10.1017/S136898002100313X)
Supplement: Supplementary file 1 [file S136898002100313Xsup001.docx]

**Supplemental Table 1**. Associations between beverage intake and actigraphy-assessed sleep characteristics in a sample of 528 Mexican youth.

|  |  | Weekend duration (minutes) | | Weekend midpoint (decimal hours) | |
| --- | --- | --- | --- | --- | --- |
|  | N | Male Adjusted Difference (95% CI)^1^ | Female Adjusted Difference | Male Adjusted Difference | Female Adjusted Difference |
| Milk |  |  |  |  |  |
| Q1 (mean=64) | 132 | Ref | Ref | Ref | Ref |
| Q2 (mean=213) | 132 | 0.6 (-28.6, 29.9) | -17.7 (-43.2, 7.8) | -0.3 (-0.7, 0.2) | 0.5 (0.1, 1.0)* |
| Q3 (mean=426) | 132 | 11.6 (-17.3, 40.5) | -6.9 (-32.6, 18.7) | -0.4 (-0.8, 0.1) | -0.01 (-0.4, 0.4) |
| Q4 (mean=738) | 132 | 6.8 (-22.2, 35.8) | -9.8 (-35.6, 16.0) | -0.0 (-0.5, 0.4) | 0.2 (-0.2, 0.6) |
| P, trend^2^ |  | 0.50 | 0.62 | 0.91 | 0.93 |
|  |  |  |  |  |  |
| Sweetened milk^3^ |  |  |  |  |  |
| Q1 (mean=0) | 505 | Ref | Ref | Ref | Ref |
| Q2 (mean=100) | 23 | -54.2 (-103.7, -4.8)* | 51.1 (7.4, 94.9)* | -0.4 (-1.2, 0.4) | -0.7 (-1.4, 0.0) |
| P, trend |  | 0.03 | 0.02 | 0.35 | 0.05 |
|  |  |  |  |  |  |
| Regular soda |  |  |  |  |  |
| Q1 (mean=9) | 132 | Ref | Ref | Ref | Ref |
| Q2 (mean=93) | 132 | 17.8 (-11.3, 47.0) | -9.1 (-33.7, 15.5) | 0.1 (-0.3, 0.6) | 0.1 (-0.3, 0.5) |
| Q3 (mean=226) | 132 | -9.1 (-38.2, 20.0) | -2.4 (-28.1, 23.3) | -0.1 (-0.5, 0.4) | 0.4 (0.0, 0.8)* |
| Q4 (mean=571) | 132 | 1.6 (-26.8, 29.9) | 6.0 (-21.9, 33.9) | 0.1 (-0.4, 0.5) | 0.5 (0.0, 0.9)* |
| P, trend |  | 0.63 | 0.64 | 0.96 | 0.02 |
|  |  |  |  |  |  |
| Coffee and tea^4^ |  |  |  |  |  |
| Q1 (mean=0) | 263 | Ref | Ref | Ref | Ref |
| Q2 (mean=59) | 133 | -10.4 (-33.6. 12.8) | -18.8 (-41.4, 3.8) | 0.2 (-0.2, 0.6) | -0.0 (-0.4, 0.3) |
| Q3 (mean=227) | 132 | 18.7 (-7.5, 44.9) | -22.8 (-44.0, 1.6) | 0.1 (-0.3, 0.5) | 0.1 (-.03, 0.4) |
| P, trend |  | 0.32 | 0.03 | 0.53 | 0.63 |
|  |  |  |  |  |  |
| Natural Fruit Juice^4^ |  |  |  |  |  |
| Q1 (mean=0) | 361 | Ref | Ref | Ref | Ref |
| Q2 (mean=40) | 84 | -10.1 (-39.0, 18.9) | -13.3 (-37.4, 10.7) | -0.5 (-0.9, 0.0) | -0.1 (-0.5, 0.3) |
| Q3 (mean=140) | 83 | 11.8 (-14.6, 38.2) | 14.0 (-12.2, 40.2) | -0.2 (-0.6, 0.2) | 0.0 (-0.4, 0.4) |
| P, trend |  | 0.53 | 0.59 | 0.16 | 0.85 |
|  |  |  |  |  |  |
| Fruit juice with added sugar |  |  |  |  |  |
| Q1 (mean=0) | 195 | Ref | Ref | Ref | Ref |
| Q2 (mean=85) | 111 | 5.9 (-21.5, 33.3) | 1.3 (-23.5, 26.0) | -0.0 (-0.5, 0.4) | 0.2 (-0.2, 0.6) |
| Q3 (mean=251) | 111 | 3.6 (-24.1, 31.2) | -12.3 (-37.0, 12.4) | 0.3 (-0.1, 0.7) | 0.3 (-0.1, 0.7) |
| Q4 (mean=630) | 111 | 11.4 (-15.3, 38.0) | -21.4 (-47.6, 4.8) | 0.3 (-0.1, 0.7) | -0.2 (-0.7, 0.2) |
| P, trend |  | 0.44 | 0.08 | 0.07 | 0.67 |
|  |  |  |  |  |  |
| Flavored water with sugar^4^ |  |  |  |  |  |
| Q1 (mean=0) | 382 | Ref | Ref | Ref | Ref |
| Q2 (mean=111) | 73 | 5.7 (-22.3, 33.8) | 7.3 (-20.2, 34.8) | -0.0 (-0.5, 0.4) | 0.2 (-0.3, 0.6) |
| Q3 (mean=494) | 73 | -13.1 (-44.4, 18.3) | -0.6 (-26.0, 24.8) | 0.0 (-0.5, 0.5) | 0.2 (-0.2, 0.6) |
| P, trend |  | 0.58 | 0.91 | 0.96 | 0.25 |
|  |  |  |  |  |  |
| Water | 195 |  |  |  |  |
| Q1 (mean=68) | 132 | Ref | Ref | Ref | Ref |
| Q2 (mean=395) | 132 | 1.6 (-27.3, 20.6) | -21.3 (-46.2, 3.6) | -0.0 (-0.5, 0.5) | 0.2 (-0.2, 0.6) |
| Q3 (mean=784) | 132 | -28.0 (-56.3, 0.4) | -5.4 (-30.5, 19.7) | 0.3 (-0.2, 0.7) | 0.2 (-0.2, 0.6) |
| Q4 (mean=1416) | 132 | -0.23 (-27.1, 26.6) | -10.8 (-37.9, 16.3) | -0.2 (-0.6, 0.3) | 0.0 (-0.4, 0.4) |
| P, trend |  | 0.59 | 0.72 | 0.63 | 0.99 |

^1^ From linear regression models with sleep characteristic as the continuous outcome and indicator variables for quantiles of beverage intake as the predictor, adjusted for sex, age, maternal education, screen time, and physical activity

^2^ P for trends are from a Wald test of a continuous variable representing ordinal categories of beverage intake quantile

^3^ Divided into a dichotomous variable due to high proportion of non-consumers

^4^ Divided into tertiles rather than quartiles due to high proportion of non-consumers

*= P for difference<0.05
